# Adenosine Receptor Ligands: Coumarin–Chalcone Hybrids as Modulating Agents on the Activity of *h*ARs

**DOI:** 10.3390/molecules25184306

**Published:** 2020-09-19

**Authors:** Saleta Vazquez-Rodriguez, Santiago Vilar, Sonja Kachler, Karl-Norbert Klotz, Eugenio Uriarte, Fernanda Borges, Maria João Matos

**Affiliations:** 1 Departamento de Química Orgánica, Facultad de Farmacia, Universidad de Santiago de Compostela, 15782 Santiago de Compostela, Spain; qosanti@yahoo.es (S.V.); eugenio.uriarte@usc.es (E.U.); 2 Institut für Pharmakologie und Toxikologie, Universität Würzburg, 97078, Würzburg, Germany; sonja.kachler@toxi.uni-wuerzburg.de (S.K.); klotz@toxi.uni-wuerzburg.de (K.-N.K.); 3 Instituto de Ciencias Químicas Aplicadas, Universidad Autónoma de Chile, 7500912 Santiago, Chile; 4 CIQUP/Department of Chemistry and Biochemistry, Faculty of Sciences, University of Porto, Rua Campo Alegre 687, 4169-007 Porto, Portugal; mfernandamborges@gmail.com

**Keywords:** coumarin, chalcone, neurodegenerative diseases, adenosine receptors, binding affinity, docking

## Abstract

Adenosine receptors (ARs) play an important role in neurological and psychiatric disorders such as Alzheimer’s disease, Parkinson’s disease, epilepsy and schizophrenia. The different subtypes of ARs and the knowledge on their densities and status are important for understanding the mechanisms underlying the pathogenesis of diseases and for developing new therapeutics. Looking for new scaffolds for selective AR ligands, coumarin–chalcone hybrids were synthesized (compounds **1**–**8**) and screened in radioligand binding (*h*A_1_, *h*A_2A_ and *h*A_3_) and adenylyl cyclase (*h*A_2B_) assays in order to evaluate their affinity for the four human AR subtypes (*h*ARs). Coumarin–chalcone hybrid has been established as a new scaffold suitable for the development of potent and selective ligands for *h*A_1_ or *h*A_3_ subtypes. In general, hydroxy-substituted hybrids showed some affinity for the *h*A_1_, while the methoxy counterparts were selective for the *h*A_3_. The most potent *h*A_1_ ligand was compound **7** (*K*_i_ = 17.7 µM), whereas compound **4** was the most potent ligand for *h*A_3_ (*K*_i_ = 2.49 µM). In addition, docking studies with *h*A_1_ and *h*A_3_ homology models were established to analyze the structure–function relationships. Results showed that the different residues located on the protein binding pocket could play an important role in ligand selectivity.

## 1. Introduction

Adenosine receptors (ARs) are cell membrane receptors, belonging to the G protein-coupled receptor (GPCRs) superfamily. ARs comprised of four different subtypes: A_1_, A_2A_, A_2B_ and A_3_ [1]. Adenosine is a purine nucleoside and an endogenous modulator of several physiological processes [1,2,3,4]. Extracellular adenosine activates the G_i_-coupled receptors of the A_1_ and A_3_ subtypes, depressing the action of the brain, heart, kidneys, and the immune system, amongst other systems, as a consequence of the inhibition of adenylyl cyclase [5]. The A_3_ subtype of AR has been cloned [6,7], making it possible to establish its pharmacological [8,9,10,11] and regulatory features [12].

Due to their widespread presence in cells, ARs proved to be promising targets in drug discovery. During the last decade, the search for selective ligands has been raised [13,14,15]. Several AR antagonists appeared as promising drug candidates for different pathological processes such as inflammation (A_3_) [14], heart and renal failure (A_1_) [16], or neurological disorders including Parkinson [17,18] and Alzheimer’s diseases (A_2A_ and/or A_1_) [19]. ARs can work as targets for various diseases and can open a new window for new therapeutic approaches.

In particular, A_1_ antagonists are effective as diuretic agents [20,21] and also show neuroprotective activity in animal models of in vivo ischemia [22]. On the other hand, A_3_ antagonists are being investigated as potential agents against renal injury [23] and also as neuroprotective agents [24,25], while A_3_ agonists are also under consideration for treating conditions of the central nervous system (CNS) and peripheral nervous system [26,27].

From the arsenal of molecules presenting high potency and selectivity on ARs, the xanthine scaffold was the first to be used to develop the so-called classical AR antagonists [28,29]. In the search for non-xanthine AR ligands, numerous structures were discovered over the years. Flavones and isoflavones have played a remarkable role. As an example, genistein, was described as a competitive antagonist at A_1_ in FRTL (thyroid) cells [30], and galangin was found to bind to the three subtypes of ARs displaying micromolar affinity for the A_3_ [31]. The affinity of flavonoids and other phytochemicals to ARs brings about the hypothesis that probably other types of natural substances, namely those present in the diet, can interact with this type of receptor.

**Figure 1:**
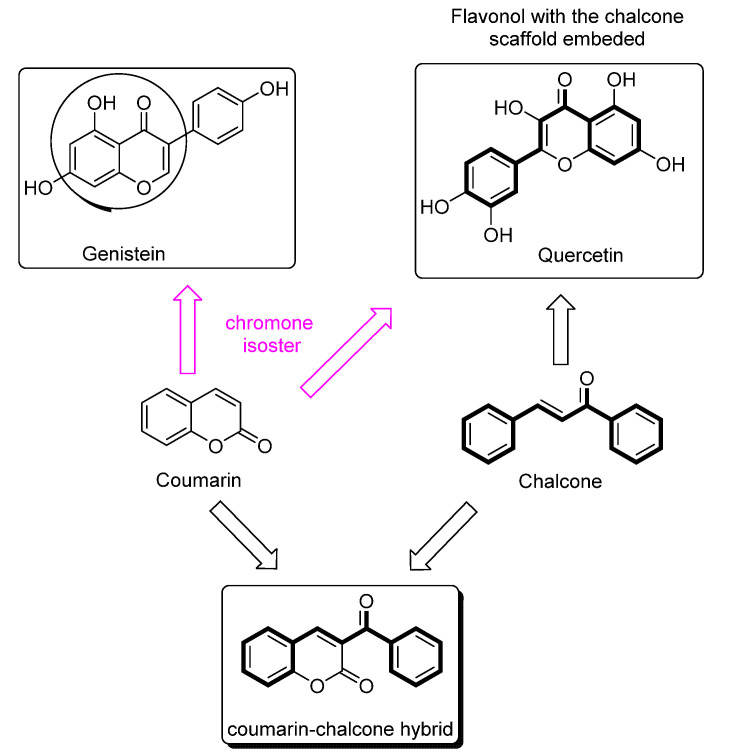
Rational design of coumarin–chalcone hybrids.

Coumarins (chromone isosteres) and chalcones (a flavonoid precursor) are naturally occurring benzopyran-related molecules presenting a variety of pharmacological activities [32,33,34]. Having in mind that both the coumarin and chalcone nuclei are structurally close to flavonoids, the design of novel AR ligands based on their scaffolds emerged as an interesting idea. Our study was also motivated by the structural similarity between the coumarin and the chromone scaffolds, which were previously described as AR ligands [35,36], and by the similarities with some coumarin derivatives previously described in our group [37,38,39,40,41,42]. In this context, we focused our attention on the 3-benzoylcoumarin core, considered as a hybrid scaffold in which the chalcone is fixed in a *trans* conformation through the double bond of the pyrone ring of the coumarin skeleton (Figure 1), presenting a more restricted conformation compared to the previously described coumarin–chalcone hybrids [36].

Therefore, based on the structural similarities between flavones, chalcones and coumarins, we decided to design and synthesize a novel family of coumarin–chalcone hybrid derivatives and study their activity on the different subtypes of human AR.

## 2. Results and Discussion

### 2.1. Chemistry

**Scheme 1:**
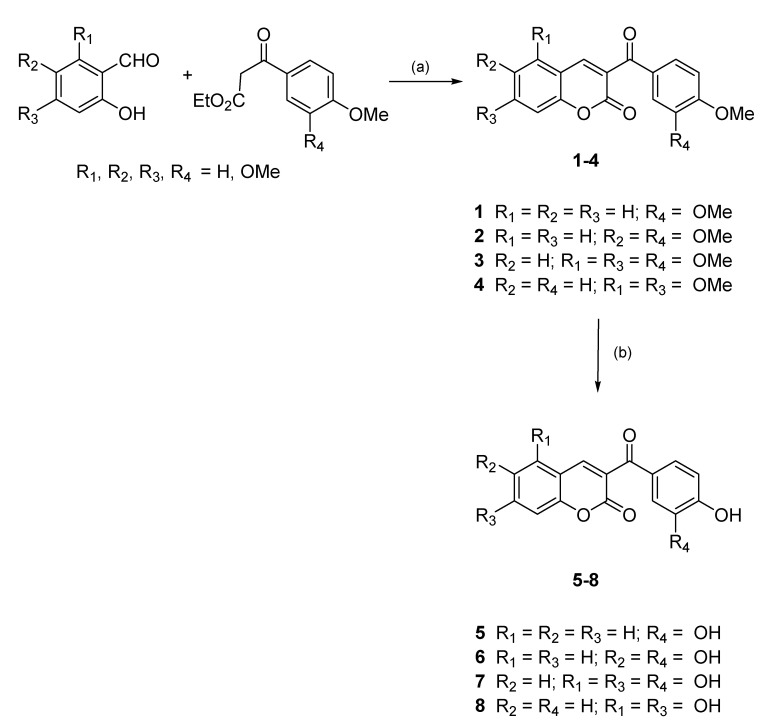
Synthetic route to obtain the coumarin-chalcone hybrids. *Reagents and conditions:* (**a**) piperidine, EtOH, reflux, 2–6 h; (**b**) BBr_3_, DCM, 80 °C, 48 h.

Two sets of coumarin–chalcone hybrids have been synthesized: one decorated with methoxy substituents (**1**–**4**) and another with hydroxy substituents (**5**–**8**). An efficient and versatile Knoevenagel reaction, treating a commercially available salicylaldehyde and the corresponding methoxylated ethyl benzoylacetate with piperidine in ethanol (EtOH) at reflux for 2–6 h, allowed the desired methoxy-3-benzoylcoumarins **1**–**4** with 85–97% yield. The hydroxy-3-benzoylcoumarins **5**–**8** were obtained by hydrolysis of the corresponding methoxy derivatives, with 75–94% yield, by employing boron tribromide (BBr_3_) as deprotecting reagent in dichloromethane (DCM) at 80 °C in a Schlenk tube for 48 h [43]. The synthetic approach is illustrated in Scheme 1 and described in the methods and materials section.

### 2.2. Pharmacology

#### Adenosine Receptor Binding Affinity Assays

The adenosine binding affinity of derivatives **1**–**8** for the human AR subtypes *h*A_1_, *h*A_2A_ and *h*A_3_, expressed in Chinese Hamster Ovary (CHO) cells, was determined in radioligand competition experiments [43,44]. In the binding affinity assay, it is measured the competition of ligands for specific binding of [^3^H]CCPA (2-chloro-*N*^6^-cyclopentyladenosine) to *h*A_1_; specific binding of [^3^H]NECA (5′-*N*-ethylcarboxamidoadenosine) to *h*A_2A_; and specific binding of [^3^H]HEMADO (2-(1-hexynyl)-*N*^6^-methyladenosine) to *h*A_3_. The results are expressed as *K*_i_ (dissociation constants), which were calculated with the program SCTFIT, and given as geometric means of at least three experiments, including 95% confidence intervals. Due to the lack of a suitable radioligand for the *h*A_2B_ receptor, the potency of antagonists at the *h*A_2B_ receptor was determined by inhibition of NECA-stimulated adenylyl cyclase activity with increasing concentrations of antagonist [43,44]. As a result, cAMP (cyclic adenosine monophosphate) production was inhibited in a concentration-dependent fashion, and K_i_ values were calculated from the measured IC_50_ values [45].

Derivatives **1**–**8** were efficiently synthesized and their in vitro binding affinity for human AR subtypes *h*A_1_, *h*A_2A_, *h*A_2B_ and *h*A_3_, expressed in CHO cells, was evaluated. In the present communication, the studies were focused on the inspection of the effect on the binding affinity of different number and position of methoxy or hydroxy substituents on the 3-benzoylcoumarin scaffold. Data obtained for the binding affinity for *h*A_1_ and *h*A_3_ is summarized in Table 1. For all the tested compounds, no significant affinity was detected for the *h*A_2A_ (*K*i > 100 µM) or *h*A_2B_ (*K*_i_ > 10 µM).

The binding affinity results show that derivatives **1** and **2**, without substitutions on the coumarin scaffold or with a single methoxy group at the position 6 of the coumarin core, respectively, display no detectable binding affinity for the evaluated receptors (*K*_i_ > 100 µM). However, the presence of two methoxy groups at positions 5 and 7 (compounds **3** and **4**, respectively) lead to an increment on both the potency and selectivity for the *h*A_3_. Compound **3**, presenting three methoxy groups at positions 5, 7 and 4′ proved to be *h*A_3_ selective, displaying a *K*_i_ = 9.03 µM, whereas compound **4**, presenting an extra methoxy groups at position 3′ is not only selective for *h*A_3_, but also displays a increase in potency (*K*_i_ = 2.49 µM). Compared to theophylline, classically used as a reference compound, we would like to highlight that both compounds **3** and **4** are more potent and *h*A_3_ selective molecules.

Based on this data, it can be concluded that both nature and position of the substitution patterns on the coumarin–chalcone scaffold play a key role in the interaction with the *h*A_3_. It can be highlighted that positions 5 and 7 of the studied scaffold seem to be relevant for the observed selectivity and potency. Analyzing the methoxylated derivatives **1**–**4**, only the molecules presenting substituents at these two positions (compounds **3** and **4**) are *h*A_3_ active and selective ligands.

Interestingly, a similar tendency was observed for *h*A_1_ binding of the hydroxylated derivatives (**5**–**8**), which bear hydroxy groups instead of methoxy groups at positions 5 and 7 (compounds **7** and **8**). Derivatives **7** and **8** display the highest potency and selectivity of the studied series towards *h*A_1_, but their activity towards this receptor is still low with *K*_i_ = 17.7 µM and *K*_i_ = 29.1 µM, respectively.

### 2.3. Theoretical Evaluation of ADME Properties

In order to explore the drug-like properties of compounds **1**–**8**, the lipophilicity, expressed as the octanol/water partition coefficient and herein named clogP, as well as other theoretical calculations such as number of hydrogen acceptors and number of hydrogen bond donors, and topological polar surface area (TPSA), were calculated using the Molinspiration software [46]. Theoretical prediction of absorption, distribution, metabolism and excretion (ADME) properties of all derivatives is summarized in Table 2.

Based on this theoretical data, it can be concluded that the study molecules **1**–**8** do not violate any of Lipinski’s rules (namely molecular weight, clogP, number of hydrogen donors and acceptors). In addition, TPSA, described as an indicator of membrane permeability, was favorable for the studied compounds.

### 2.4. Molecular Modeling

*h*A_1_ and *h*A_3_ homology models were successfully constructed (Materials and methods section). A selection of models obtained from Induce Fit calculations were tested based on their ability to discriminate between known ligands, decoys and between subtype-selective compounds. The models selected for the docking calculations showed excellent results in both tests. A dataset of 200 randomly selected decoys from the ZINC database [47] were mixed up with 22 known ligands of each adenosine receptor subtype [48] Glide SP precision was used to dock the database to the *h*A_1_ and *h*A_3_ models [49]. Table 3 presents the area under the receiver operating characteristic (ROC) curve (AUROC) for both systems. To differentiate between subtype-selective ligands, a second and more challenging test was performed. As in a previous study [48], 66 subtype-selective molecules (22 *h*A_1_, 22 *h*A_2A_ and 22 *h*A_3_ compounds) were docked to the *h*A_1_ model (22 true positives vs. 44 false positives) and to the *h*A_3_ (22 true positives vs. 44 false positives). Results corroborate those previously published by Katritch et al. [50] and proved that the developed homology models are able to discriminate between subtype-selective compounds (Table 3).

**Figure 2:**
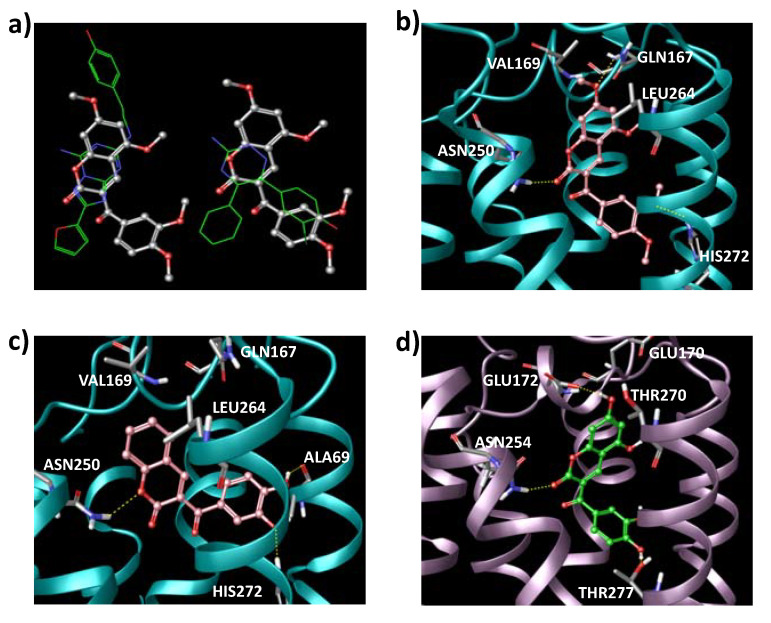
(**a**) Comparative study of the co-crystallized ligands (green carbons) in the *h*A_2A_ [3EML (left) and 3UZC (right)] with the pose of compound **3** extracted from the *h*A_3_ docking calculations (grey carbons). Binding pockets in *h*A_2A_ and *h*A_3_ were superposed. (**b**) Pose extracted for compound **3** inside the *h*A_3_ after docking. Hydrogen bonds are represented in yellow color. (**c**) Hypothetical binding mode for compound **5** (pink carbons) in the *h*A_3_. (**d**) Pose obtained through docking simulations for compound **7** (green carbons) in the *h*A_1_ protein pocket.

Glide SP molecular docking simulations were run with our data using the *h*A_1_ and *h*A_3_ selected homology models as protein structures to detect the hypothetical binding mode of the new synthesized compounds [51]. The Prime module was used to optimize the protein structure for each binding mode [52]. Molecular docking simulations are represented in Figure 2.

Docking calculations and the established homology models for the *h*A_1_ and *h*A_3_ identified the hypothetical binding mode and rationalized the interaction of these derivatives with their respective ARs binding sites.

The calculations showed a high level of variability since all the synthetized derivatives yielded different possible binding modes inside the pockets. Selection of the hypothetical binding pose was accomplished considering the number of similar poses extracted from the simulations and geometrical correspondence to crystallized ligands in the *h*A_2A_ (Figure 2a).

Docking results disclosed important data about the binding mode: the oxygens presented in the benzopyrone system are oriented towards the Asn250 residue and the benzoyl moiety was buried in the *h*A_3_ pocket. This hypothetical binding mode corroborates the conformations shown by the co-crystallized ligands in the *h*A_2A_ (PDB: 3EML and 3UZC) [48,53] (Figure 2a,b). The pose of compound **3** produced effective hydrogen bonds with Gln167, Asn250 and His272 residues.

Interestingly, when methoxy substituents were demethylated and changed into hydroxy equivalents (compounds **5**–**8**) a modification in the profile of the studied derivatives was noticed: a loss of affinity for *h*A_3_ and a tendency for interaction with *h*A_1_. The only compound that discloses some affinity for both receptors was compound **5** (*h*A_1_ *K*_i_ = 39.5 and *h*A_3_ *K*_i_ = 34.5 µM), which presents a catechol at positions 3′ and 4′ and no substitutions in the coumarin fragment. The hypothetical binding mode for compound **5** in the *h*A_3_ pocket is represented in Figure 2c. The compound can establish hydrogen bonds with Ala69, Asn250 and His272 residues. As observed in the *h*A_2A_ crystallized structure and previously published studies [54,55], the corresponding Asn250 residue seems to play an important role in ligand recognition. The compound **5** pose inside the *h*A_1_ pocket is likewise the described pose in the *h*A_3_ one. However, the position was slightly shifted, and calculations were not able to retrieve a hydrogen bond with the Asn250 residue. The introduction of an additional hydroxy group at position 6 of the coumarin scaffold (compound **6**), resulted in a loss of measurable *h*A_3_ binding affinity. The most noticeable binding affinities were found for derivatives with hydroxy substitutions at positions 5 and 7 of the coumarin core, as stated for methoxy equivalents. Thereby, compound **7**, with the same substitution pattern as quercetin (Figure 1), that is, hydroxy groups at positions 5, 7, 3′ and 4′, displays *h*A_1_ selectivity, and the best binding affinity (*K*_i_ = 17.7 µM). Compound **8**, with the same substitution pattern as genistein (Figure 1, hydroxy substituents at positions 5, 7 and 4′) shows a similar *h*A_1_ selectivity (*K*_i_ = 29.1 µM). The pose obtained through docking calculations for compound **7** in the *h*A_1_ protein pocket showed the possibility of establishment of hydrogen bonds with Glu172, Asn254 and Thr277 residues (Figure 2d).

**Figure 3:**
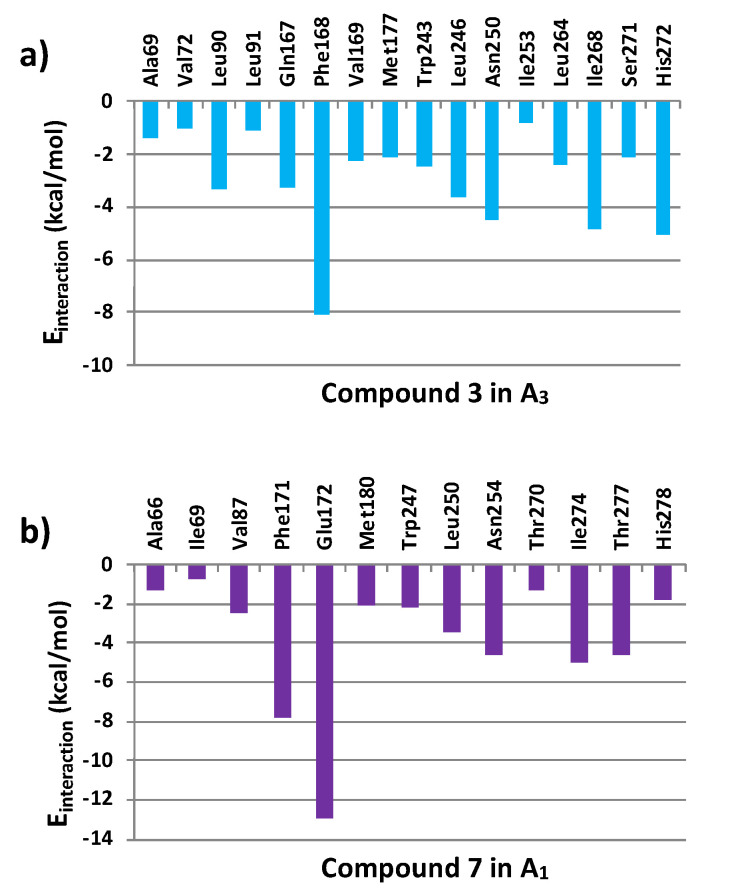
Interaction energy contribution (sum of Coulomb, *van der Waals* and hydrogen bond energies) between the residues in the (**a**) *h*A_3_ and (**b**) *h*A_1_ and the respective derivatives **3** and **7** (residues in a distance of 3 Å from the ligand).

Moreover, we calculated the interaction energy contributions of the residues in *h*A_3_ and *h*A_1_ pockets with compounds **3** and **7**, respectively (Figure 3). The sum of different individual contributions, such as Coulomb, *van der Waals* and hydrogen bond energies, was taken into account in the calculation of the interaction energies for each residue.

**Figure 4:**
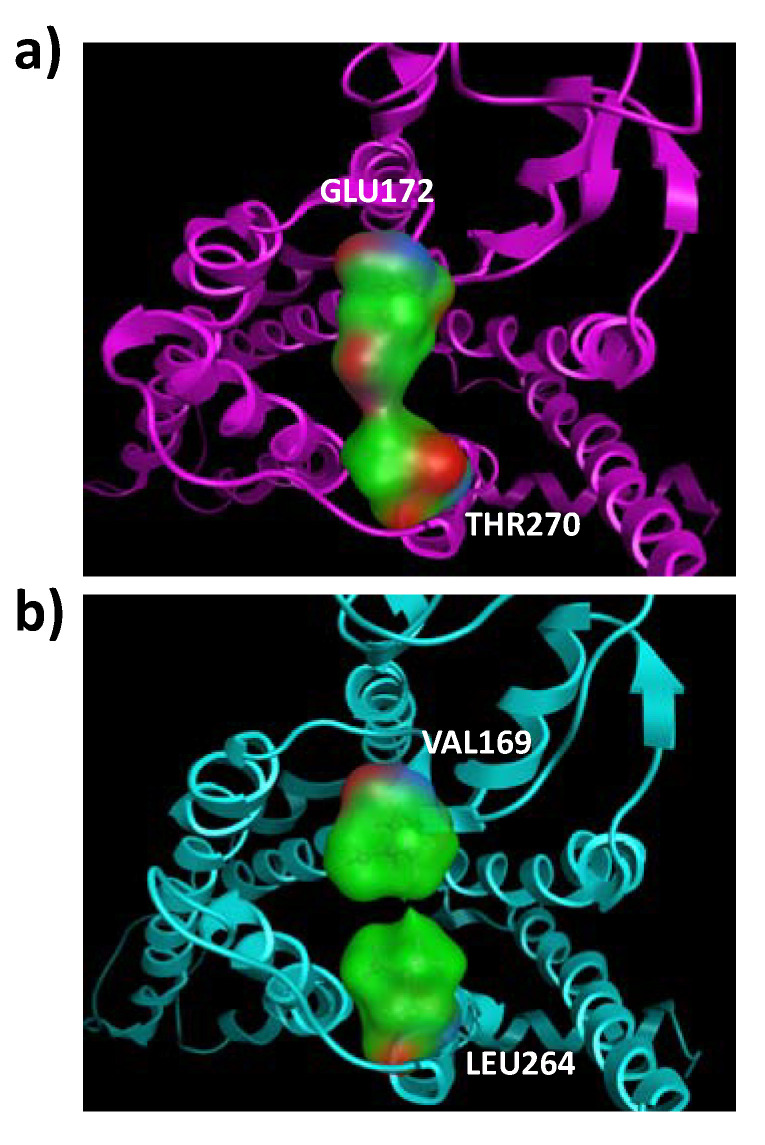
Molecular surface showing favored interaction areas generated in the (**a**) *h*A_1_ and (**b**) *h*A_3_. Red color represents hydrogen-bond areas, green color shows hydrophobic areas, and blue represents mildly polar interfaces. Protein structures are viewed from the extracellular side.

In addition, Figure 4 shows the molecular surface around the two residues in the *h*A_1_ and *h*A_3_ that could be responsible for the observed selectivity.

Regarding the interaction energy contributions (Figure 4), calculations showed that the molecular surface around the two residues in the *h*A_1_ and *h*A_3_ could be responsible for the observed selectivity. Phe168, Asn250, Ile268 and His272 are important residues in the interaction between compound **3** and the *h*A_3_. Residues with important contributions in the stabilization of compound **7** inside the *h*A_1_ are Phe171, Gln172, Asn254, Ile274 and Thr277.

There are different residues in both *h*A_1_ and *h*A_3_ with different hydrophobic/hydrophilic characteristics, which may be important to understand the observed selectivity. Hydrophobic residues in the *h*A_3_, such as Val169 and Leu264, could establish hydrophobic interactions and contribute towards stabilizing the ligand when the derivatives present hydrophobic substituents, like methoxy groups (i.e., **3** and **4**) (Figure 4). However, in the case of *h*A_1_, the corresponding residues are Glu172 and Thr270. They have hydrophilic characteristics and so can stabilize the binding of derivatives with polar substituents, such as the hybrids with hydroxy groups (compounds **6**–**8**). Yet, compound **5,** with no substituents in the coumarin ring, can be stabilized in the pocket of both proteins.

## 3. Materials and Methods

### 3.1. Chemistry

#### 3.1.1. General Methods

Starting materials and reagents were obtained from commercial suppliers and were used without purification. Melting points (mp) were determined using a Reichert Kofler thermopan or in capillary tubes on a Büchi 510 (Flawil, Switzerland) apparatus and were uncorrected. ^1^H-NMR (300 MHz) and ^13^C-NMR (75.4 MHz) spectra were recorded with a Bruker AMX spectrometer (Bruker Daltonics Inc., Fremont, CA, USA) using DMSO-*d_6_* or CDCl_3_ as solvent. Chemical shifts (δ) are expressed in parts per million (ppm) using TMS as an internal standard. Coupling constants *J* are expressed in hertz (Hz). Spin multiplicities are given as s (singlet), bs (broad singlet), d (doublet), dd (doublet of doublets) and m (multiplet). Mass spectrometry was carried out with a Kratos MS-50 or a Varian MAT-711 spectrometer (Thermo Fisher Scientific, Waltham, MA, USA). Elemental analyses were performed by a Perkin–Elmer 240B microanalyzer (Thermo Fisher Scientific, Waltham, MA, USA) and were within ±0.4% of the calculated values in all cases. The analytical results were ≥95% purity for all compounds. Flash Chromatography (FC) was performed on silica gel (Merck 60, 230–400 mesh, Kenilworth, NJ, USA) and analytical TLC on precoated silica gel plates (Merck 60 F254, Kenilworth, NJ, USA). Organic solutions were dried over anhydrous sodium sulfate. Concentration and evaporation of the solvent after reaction or extraction was carried out on a Büchi rotavapor (BÜCHI Labortechnik AG, Switzerland) operating at reduced pressure. The purity of compounds was assessed by high performance liquid chromatography (HPLC) coupled at diode array detector (DAD) on a Thermo Quest Spectrasystem (Thermo Fisher Scientific, Waltham, MA, USA) equipped with a P4000 pump, an UV6000 UV-Vis diode array detector, and a SN4000 interface to be operated via a personal computer. The instrument software ChromQuest 5.0 (Thermo Fisher Scientific, Waltham, MA, USA) was used for data acquisition. Different analytical columns and mobile phases (all solvents were HPLC grade) were tested. The mobile phase was H_2_O:CH_3_CN = 70:30 and an Eclipse xdb C18 column (5 μm particle size, 0.46 mm i.d., 25 cm length; Agilent Technologies, CA, USA) was used. The purity of the compounds was found to be higher than 95%.

#### 3.1.2. Synthetic Protocol to Obtain the Methoxy-3-benzoylcoumarins **1**–**4**

To a solution of the appropriate *β*-ketoester (1 mmol) and the corresponding salicylaldehyde (1 mmol) in ethanol (5 mL) piperidine in catalytic amount (0.10 mL) was added. The reaction mixture was refluxed for 2–6 h and, after completion (followed by TLC), the reaction was cooled, and the precipitate was filtered and washed with cold ethanol and ether. The obtained solid was recrystallized in DCM to afford the corresponding methoxy-3-benzoylcoumarin compounds.

#### 3.1.3. Synthetic Protocol to Obtain the Hydroxy-3-benzoylcoumarins **5**–**8**

In a Schlenk tube, the appropriate methoxy derivative compound **1**–**4** (1 mmol) was dissolved in DCM (1 mL), and BBr_3_ (20 mmol, 1M) was added dropwise. The tube was sealed, and the reaction mixture was heated at 80 °C for 48 h. The resulting crude product was treated with MeOH and rotated to dryness. The obtained crude solid was recrystallized in MeOH or purified by flash chromatography using hexane/ethyl acetate mixtures as eluent, to afford the desired hydroxy derivatives.

3-(3′,4′-Dimethoxybenzoyl)coumarin (**1**): 85% yield; white solid; mp 190–191 °C; ^1^H-NMR (300 MHz, CDCl_3_) δ ppm 8.01 (s, 1H, H-4), 7.71–7.52 (m, 3H, 3x Ar-H), 7.50–7.29 (m, 3H, 3x Ar-H), 6.87 (d, *J* = 8.4 Hz, 1H, H-5′), 3.95 (s, 6H, 2x OCH_3_); ^13^C-NMR (75 MHz, CDCl_3_) δ ppm 190.3, 154.8, 154.4, 149.5, 144.6, 133.6, 129.3, 129.2, 127.8, 125.7, 125.2, 118.5, 117.2, 111.2, 110.2, 56.4, 56.3; EI-MS *m*/*z* (%): 311 ([M + 1]^+^, 59), 310 (M^+^, 100), 173 (41), 166 (25), 165 (99), 79 (22), 77 (22); Anal. Calcd. For C_18_H_14_O_5_: C 69.67, H 4.55. Found: C 69.69, H 4.58.

6-Methoxy-3-(3′,4′-dimethoxybenzoyl)coumarin (**2**): 97% yield; beige solid; mp 202–203 °C; ^1^H-NMR (300 MHz, CDCl_3_) δ ppm 7.78 (s, 1H, H-4), 7.38 (d, *J* = 1.9 Hz, 1H, H-2′), 7.26 (dd, *J* = 8.4, 2.0 Hz, 1H, H-6′), 7.16 (d, *J* = 9.1 Hz, 1H, H-8), 7.04 (dd, *J* = 9.1, 2.9 Hz, 1H, H-7), 6.82 (d, *J* = 2.9 Hz, 1H, H-5), 6.70 (d, *J* = 8.4 Hz, 1H, H-5′), 3.78 (s, 6H, 2x OCH_3_), 3.69 (s, 3H, OCH_3_); ^13^C-NMR (75 MHz, CDCl_3_) δ ppm 190.4, 156.6, 154.4, 149.5, 149.3, 144.4, 129.4, 128.0, 125.7, 121.6, 118.8, 118.2, 111.2, 110.8, 110.2, 56.4, 56.3, 56.1; EI-MS *m*/*z* (%): 341 ([M + 1]^+^, 58), 340 ([M]^+^, 94), 165 (100), 77 (22); Anal. Calcd. For C_19_H_16_O_6_: C 67.05, H 4.74. Found: C 67.09, H 4.75.

5,7-Dimethoxy-3-(3′,4′-dimethoxybenzoyl)coumarin (**3**): 91% yield; pale yellow solid; mp 210–211 °C; ^1^H-NMR (300 MHz, CDCl_3_) δ ppm 8.19 (s, 1H, H-4), 7.34 (d, *J* = 1.9 Hz, 1H, H-2′), 7.26 (dd, *J* = 8.4, 2.0 Hz, 1H, H-6′), 6.70 (d, *J* = 8.4 Hz, 1H, H-5′), 6.29 (d, *J* = 2.0 Hz, 1H, H-6), 6.14 (d, *J* = 2.0 Hz, 1H, H-8), 3.77 (bs, 6H, 2x OCH_3_), 3.72 (bs, 6H, 2x OCH_3_); ^13^C-NMR (75 MHz, CDCl_3_) δ ppm 190.9, 165.8, 159.4, 158.4, 158.0, 153.9, 149.3, 141.5, 130.0, 125.4, 121.3, 111.5, 110.1, 103.9, 95.4, 93.0, 56.3; EI-MS *m*/*z* (%): 371 ([M + 1]^+^, 24), 370 (M^+^, 100), 339 (21), 233 (30), 165 (63); Anal. Calcd. For C_20_H_18_O_7_: C 64.86, H 4.90. Found: C 64.88, H 4.93.

5,7-Dimethoxy-3-(4′-methoxybenzoyl)coumarin (**4**): 97% yield; pale yellow solid; mp 174–175 °C; ^1^H-NMR (300 MHz, CDCl_3_) δ ppm 8.21 (s, 1H, H-4), 7.69 (d, *J* = 8.8 Hz, 2H, H-2′, H-6′), 6.77 (d, *J* = 8.8 Hz, 2H, H-3′, H-5′), 6.29 (d, *J* = 2.2 Hz, 1H, H-6), 6.13 (d, *J* = 2.2 Hz, 1H, H-8), 3.72 (2s, 3H + 3H, 2x OCH_3_), 3.70 (s, 3H, OCH_3_); ^13^C-NMR (75 MHz, CDCl_3_) δ ppm 190.7, 165.6, 163.8, 159.1, 158.2, 157.8, 141.5, 132.1, 129.8, 121.2, 113.7, 103.8, 95.2, 92.8, 56.1, 56.0, 55.5; EI-MS *m*/*z* (%): 341 ([M + 1]^+^, 33), 340 (M^+^, 88), 325 (28) 312 (30), 309 (45), 297 (20), 233 (48), 135 (100), 92 (27), 77 (38). Anal. Calcd. For C_19_H_16_O_6_: C 67.05, H 4.74. Found: C 67.08, H 4.76.

5,7-Dihydroxy-3-(4′-hydroxybenzoyl)coumarin (**8**): 88% yield; pale green solid; mp 290–292 °C; ^1^H-NMR (300 MHz, DMSO-*d*_6_) δ ppm 11.10 (s, 1H), 10.85 (s, 1H), 10.53 (s, 1H), 8.09 (d, *J* = 1.4 Hz, 1H), 7.67 (d, *J* = 8.6 Hz, 2H), 6.80 (d, *J* = 8.7 Hz, 2H), 6.25 (d, *J* = 2.0 Hz, 1H), 6.22 (d, *J* = 1.8 Hz, 1H); ^13^C-NMR (75 MHz, DMSO-*d*_6_) δ ppm 190.4, 164.2, 162.4, 158.8, 157.5, 157.2, 141.1, 132.3, 128.3, 119.0, 115.3, 101.5, 98.5, 94.3. EI-MS *m*/*z* (%): 299 ([M + 1]^+^, 9), 298 (M^+^, 31), 283 (16), 218 (20), 121 (100), 93 (26), 65 (27). Anal. Calcd. For C_16_H_10_O_6_: C 64.43, H 3.38. Found: C 64.39, H 3.37.

### 3.2. Biological Assays

#### 3.2.1. Binding Affinity Assays

The binding affinity for *h*A_1_, *h*A_2A_, *h*A_3_ of the synthetized compounds was evaluated using radioligand competition experiments in CHO cells that were stably transfected with the individual receptor subtypes [44,45]. The radioligands used were 1 nM [^3^H]CCPA for *h*A_1_ (*K*_D_ = 0.61 nM), 10 nM [^3^H]NECA for *h*A_2A_ (*K*_D_ = 20.1 nM), and 1 nM [^3^H]HEMADO for *h*A_3_ (*K*_D_ = 1.2 nM) receptors. Due to the lack of a suitable radioligand for the *h*A_2B_ receptor, the potency of antagonists at the *h*A_2B_ receptor (expressed on CHO cells) was determined by inhibition of NECA-stimulated adenylyl cyclase activity [44,45]. The IC_50_ for inhibition of cAMP (cyclic adenosine monophosphate) production was determined and converted to K_i_ values using the Cheng and Prusoff equation [56]. For all the tested compounds, no measurable activity for the *h*A_2B_ (K_i_ > 10 µM) was detected.

#### 3.2.2. Statistical Methods

*K*_i_ values (dissociation constants) were determined in radioligand competition experiments with 7–8 different concentrations of test compound and each concentration was tested in duplicate. *K*_i_ values are given as geometric means of three independent experiments with 95% confidence intervals. The program Prism 6 (GraphPad Software) was used for the analysis of the competition curves.

### 3.3. Theoretical Evaluation of ADME Properties

cLogP was calculated by the methodology developed by Molinspiration as a sum of fragment-based contributions and correction factors. Topological Polar Surface Area (TPSA) was calculated based on the methodology published by Ertl et al. as a sum of fragment contributions [57]. Oxygen- and nitrogen-centered polar fragments are considered. TPSA has been shown to be a very good descriptor characterizing drug absorption, including intestinal absorption, bioavailability, Caco-2 permeability and blood–brain barrier penetration. The method for calculation of molecule volume developed at Molinspiration is based on group contributions. These have been obtained by fitting the sum of fragment contributions to “real” 3D volume for a training set of about twelve thousand, mostly drug-like molecules. Three-dimensional molecular geometries for a training set were fully optimized by the semiempirical AM1 method.

### 3.4. Molecular Modeling

Homology modeling was carried out using the Molecular Operating Environment (MOE) suite [49]. Molecular docking simulations were performed with the Schrodinger package [51,52].

#### 3.4.1. Homology Models of *h*A_1_ and *h*A_3_

Homology models of the *h*A1 and *h*A_3_ were constructed. The crystallized structure of the *h*A_2A_ receptor (PDB: 3EML) was used as a template [48]. Protein sequence alignment of the 3 receptors (*h*A_1_, *h*A_2A_ and *h*A_3_) used to generate the homology models was performed as previously described by Katritch et al. [50]. The alignment was made considering the highly conserved residues in the different TM helices. MOE software was used to generate the homology models [49]. Protein geometry was evaluated for the models taking into account Phi–Psi plots, rotamers, bond angles, bond lengths, atom clashes, dihedrals and contact energies. The presence of different conserved disulfide bridges was manually checked, such as the bridge between the corresponding Cys77 and Cys166 residues in the *h*A_2A_. Induce Fit Docking Workflow in the Schrodinger package was used to optimize the final models [58]. Selective high affinity ligands (compounds coll_11 and jaco_mre3008_f20) [50] were used to adapt the protein pocket for the *h*A_1_ and *h*A_3_, respectively. This procedure involved three steps: 1) Glide-based docking of the ligands using SP mode (standard-precision); 2) Protein pocket optimization using Prime and considering the residues within 5Å from the ligand poses; 3) Glide-based docking of the ligands in the refined pocket using XP mode (Extra-precision). As previously described [50], homology models were tested for their capability to discriminate ligands from decoys and between known subtype-selective compounds. ROC curves were performed, and the best models were selected for further molecular docking studies.

#### 3.4.2. Molecular Docking of *h*A_1_ and *h*A_3_ ARs

Molecular docking studies using the *h*A_1_ and *h*A_3_ homology models, selected in the previous step, were carried out. Compounds were docked using Glide SP mode [52]. Ten poses for each ligand were collected and optimized using MM-GBSA in Prime [53], taking into account a flexible protein region defined by 5 Å from the ligand. Final binding modes were selected, taking into account the number of similar poses extracted from the calculations and geometrical correspondence to co-crystallized ligands in the *h*A_2A_.

## 4. Conclusions

The current study was focused on the synthesis and the evaluation of binding affinity towards the four subtypes of human ARs of a selected series of methoxy and hydroxy coumarin–chalcone hybrids. Structure–activity relationship (SAR) studies of the new molecules highlighted that, in general, methoxy substitutions, as in the example of compounds **3** and **4,** allow a superior *h*A_3_ binding affinity and selectivity, whereas the hydroxy substitutions, as in the example of compounds **5**–**8**, allow a modest *h*A_1_ binding affinity. Substitutions at positions 5 and 7 of the coumarin scaffold proved to be essential for the potency and selectivity in both series of compounds. Compound **4**, a methoxy derivative, and compound **7**, a hydroxy derivative, proved to be the most potent compounds of the studied series, displaying a *h*A_3_ *K*_i_ = 2.49 µM and a *h*A_1_ *K*_i_ = 17.7 µM, respectively. Docking calculations allow an understanding the binding preference of the studied molecules. Finally, the theoretical values for the ADME properties show that all the coumarin–chalcone hybrids **1**–**8** do not break any of Lipinski’s rules, being promising scaffolds for further compound optimization.

## Figures and Tables

**Table 1:** Binding affinity (*K_i_*) of compounds **1**–**8** on *h*A_1_ and *h*A_3_ AR.

Compound	*h*A_1_ (*K_i_* µM) ^a^	*h*A_3_ (*K_i_* µM) ^a^
**1**	>100	>100
**2**	>100	>100
**3**	>30 ^b^	9.03 (6.28–13.0)
**4**	>100	2.49 (2.33–2.66)
**5**	39.5 (25.3–61.5)	34.5 (29.7–40.1)
**6**	54.0 (49.8–58.5)	>60 ^b^
**7**	17.7 (16.0–19.5)	>30 ^b^
**8**	29.1 (20.4–41.5)	>60 ^b^
Theophylline	6.77 (4.07–11.3)	86.4 (73.6−101)

^a^ Results are geometric means of 3 experiments and given with 95% confidence intervals (in parentheses). ^b^ At higher concentrations, the compounds precipitate.

**Table 2:** Theoretical evaluation of the ADME properties of coumarin–chalcone hybrids.^a.^

Compound	clogP	TPSA (Å^2^)	*n*-OH Acceptors	*n*-OHNH Donors	Volume (Å^3^)
**1**	3.04	65.75	5	0	270.07
**2**	3.08	74.98	6	0	295.62
**3**	3.06	84.22	7	0	321.16
**4**	3.47	74.98	6	0	295.16
**5**	2.43	87.74	5	2	235.01
**6**	1.93	107.97	6	3	243.03
**7**	1.63	128.20	7	4	251.05
**8**	2.12	107.97	6	3	243.03

^a^ TPSA, topological polar surface area; n-OH, number of hydrogen acceptors; n-OHNH, number of hydrogen bond donors.

**Table 3:** Area under the ROC curve (AUROC) for the two homology models.

AUROC	*h*A_1_	*h*A_3_
test 1 ^a^	0.91	0.95
test 2 ^b^	0.86	0.82

^a^ 22 *h*A_1_ or 22 *h*A_3_ ligands as true positives (TP) and 200 random decoys as false positives (FP) were considered. ^b^ For *h*A_1_, 22 *h*A_1_ selective compounds as TP and 22 *h*A_2A_ + 22 *h*A_3_ compounds as FP were considered. For *h*A_3_, 22 *h*A_3_ compounds as TP and 22 *h*A_2A_ + 22 *h*A_1_ compounds as FP were considered.
